# Retromolar intubation for the general anesthesia of maxillofacial fracture patients

**DOI:** 10.1186/s12903-024-04583-1

**Published:** 2024-07-15

**Authors:** Xiaorong Tang, Lili Ding, Yitao Li

**Affiliations:** Department of Dentistry, Nantong First People’s Hospital, Nantong, 226001 Jiangsu China

**Keywords:** Retromolar intubation, General anesthesia, Endotracheal tube, Maxillofacial fracture

## Abstract

**Background:**

Evaluate the possibility of retromolar intubation for general anesthesia in patients with maxillofacial fractures.

**Methods:**

The medical records of 54 patients with maxillofacial fractures who visited the Oral and Maxillofacial Surgery Department of Nantong First People’s Hospital from January 2020 to August 2022 were collected. The retromolar areas of each patient were measured from the coronal CT images, and correlated with the patient’s age, sex, type of fracture (i.e., maxillary fracture, mandibular fracture, or complex fracture of multiple maxillofacial bones), and the presence of the third molar (verified from 3D CT). The dimensions of the retromolar areas were finally compared with the outer diameter (OD) of standard endotracheal tubes (ETTs), most importantly the size 7.5 ETT (OD 10.3 mm) for male and the size 7.0 ETT (OD 9.8 mm) for female.

**Results:**

The survey included 38 male and 16 female patients, with an average age of 44.1 and 54.3 years, respectively. The dimensions of the retromolar area (height × width) were as follows: male, (9.39 ± 1.77) mm × (12.08 ± 0.98) mm on the left and (9.81 ± 2.23) mm × (11.77 ± 1.08) mm on the right; female, (8.82 ± 1.53) mm × (10.51 ± 1.00) mm on the left and (9.73 ± 1.60) mm × (10.63 ± 1.58) mm on the right. The width was always larger than the OD of the routinely used ETT, but the height could be smaller by less than 1 mm. However, the oral mucosa can be compressed to allow the ETT to fit in the retromolar area.

**Conclusions:**

The retromolar area provided appropriate space to place a reinforced ETT for patients with maxillofacial fractures needing general anesthesia that must not interfere with intermaxillary ligation. Retromolar intubation can help maxillofacial fracture surgeries that focus on occlusal restoration.

**Supplementary Information:**

The online version contains supplementary material available at 10.1186/s12903-024-04583-1.

## Background

Restoring occlusal contact is the primary objective in treating patients with maxillofacial fractures, and surgery is usually performed under general anesthesia with endotracheal intubation. Whereas the general anesthesia for typical surgeries can be readily performed via oral or nasal endotracheal intubation, for which airway management is accomplished with a cuffed anesthesia catheter, the situation is much more complicated for patients with craniomaxillofacial trauma. For example, routine oral intubation is not possible when intraoperative intermaxillary ligation is planned to restore occlusion [1], at which point nasal intubation may still be considered, but invasive approaches such as tracheostomy or submental intubation seem to be the last resort if there are also contraindications for nasal intubation. Tracheal intubation through retromolar area was first reported by Bonfil in 1983 [2] and later detailed by Martinez-Lage et al. in 1998 [3]. Based on the success from 39 surgeries, Martinez-Lage et al. called for the extraction of the third molar and a semilunar osteotomy at the retromolar area, for the endotracheal tube (ETT) to fit in the resulting notch and not disrupt the alignment of the upper and lower molars, essentially implying that the retromolar area is not big enough to accommodate a suitably sized ETT for appropriate ventilation, although they did briefly comment that retromolar intubation could be performed without osteotomy for a few patients. However, subsequent studies that measured healthy subjects’ rotational panoramic radiography [4] or three-dimensional computed tomography (3D CT) [5] contended that the retromolar area is not as narrow as previously believed and carrying out retromolar intubation completely non-invasively is possible.

### Case reports

This study was inspired by the success of the two clinical cases described in the following. This study was approved by the Ethics Committee of Nantong First People’s Hospital (No. 2024KT066).

Patient 1 was a 54-years-old female who sustained open zygomaticomaxillary fracture, nasal fracture, orbital floor fracture, and skull base fracture due to a car accident. She was 165 cm and 55 kg, and her body mass index (BMI) was 23.9. The planned surgical treatment was open reduction and internal fixation of maxillary fracture along with closed reduction of nasal fracture under general anesthesia. Nasal intubation was prohibited by both the skull base fracture (an absolute contraindication) and the planned nasal surgery. In addition, the patient refused tracheostomy or submental intubation. Preoperative 3D reconstruction of maxillofacial features and coronal CT showed that all third molars except the one on the lower left were present and erupted.

**Fig. 1:**
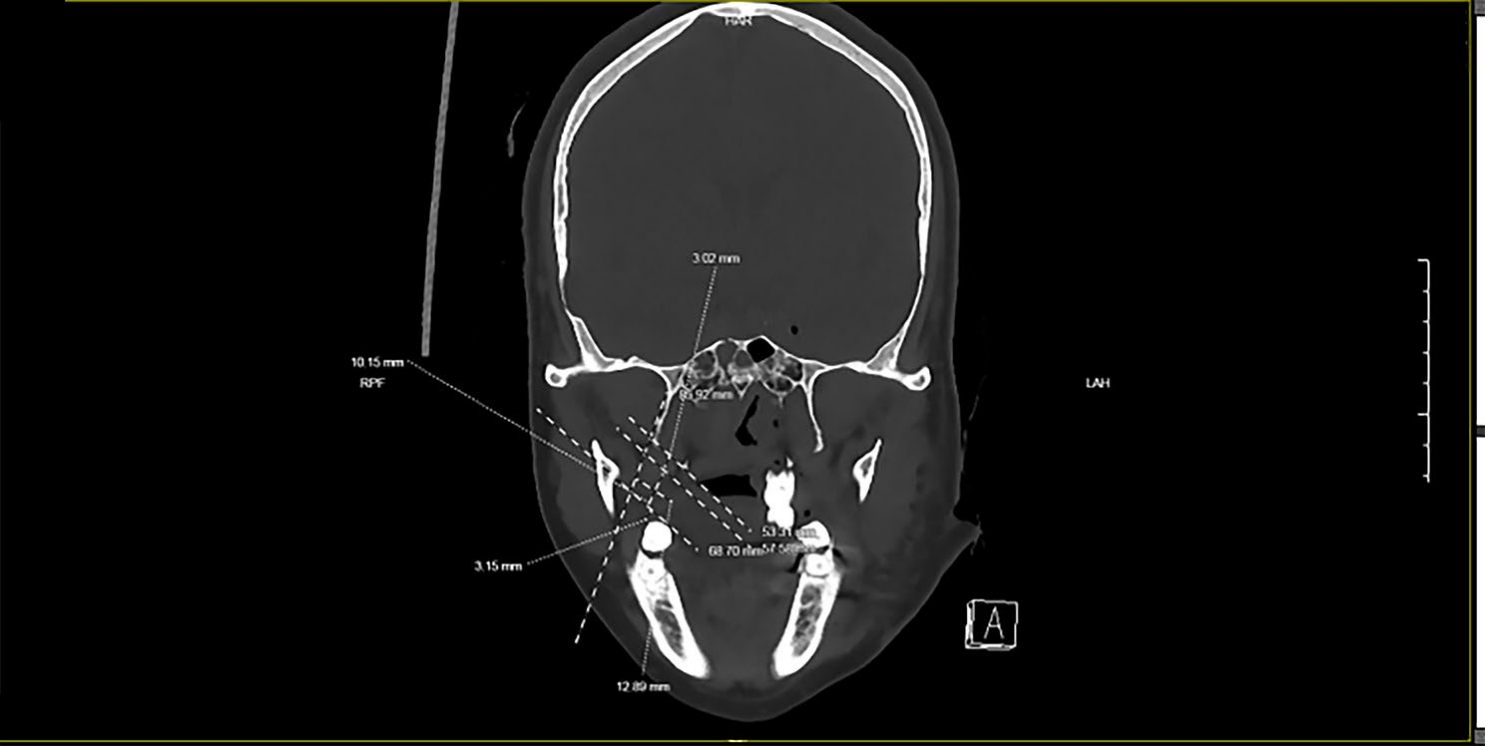
Measurement of the retromolar area from 3D facial reconstruction

Figure 1 illustrates how the dimensions of the retromolar areas were measured from 3D facial reconstruction. The section closest to the distal aspect of the last molar was selected as the reference plane. The height of the retromolar area was measured as the distance, along the axis of the lateral margin of maxilla, from 3 mm below the bone edge of the maxillary alveolar bone to 3 mm above the bony edge of the mandible. A line perpendicular to the measured height and passing its midpoint was drawn from the lingual margin to the lateral margin of maxilla, and its length was taken as the width of the retromolar area. The average values from three measurements were used. Her retromolar areas (height × width) were 11.60 mm × 12.84 mm on the left side and 11.00 mm × 14.71 mm on the right side.

**Fig. 2:**
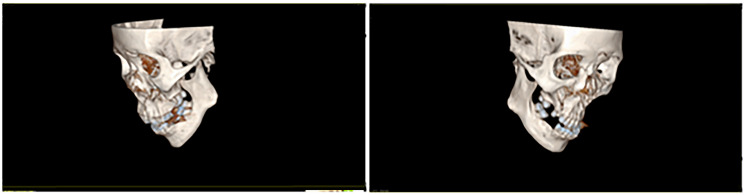
Patient 2’s preoperative 3D reconstruction

Patient 2 was a 20-years-old male whose left mandible got fractured in a car accident. He was 172 cm and 59 kg, and his BMI was 19.9. Nasal intubation was ruled out due to nasal cavity deformity. Preoperative 3D reconstruction of maxillofacial features and coronal CT showed that the upper right third molar was the only one remaining and it had erupted (Fig. 2). Based on the average of three measurements, the retromolar areas (height × width) were 11.73 mm × 13.65 mm on the left side and 12.72 mm × 12.95 mm on the right side.

**Fig. 3:**
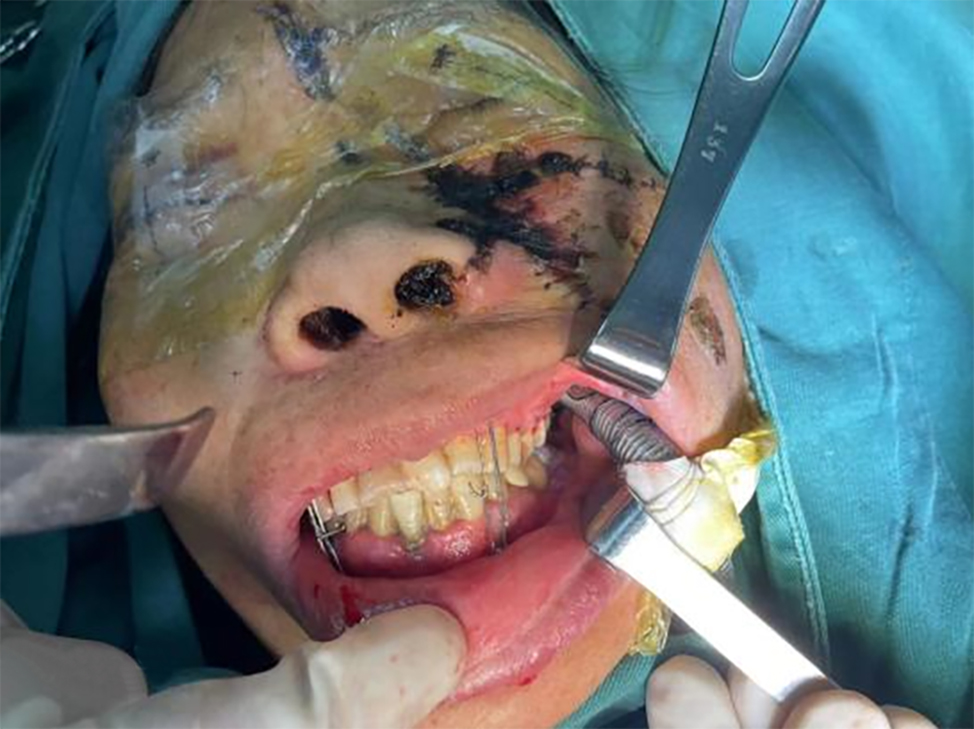
The ETT was pushed back and bent to fit in the left retromolar area and exit from the left corner of the mouth before intermaxillary fixation (Patient 1)

**Fig. 4:**
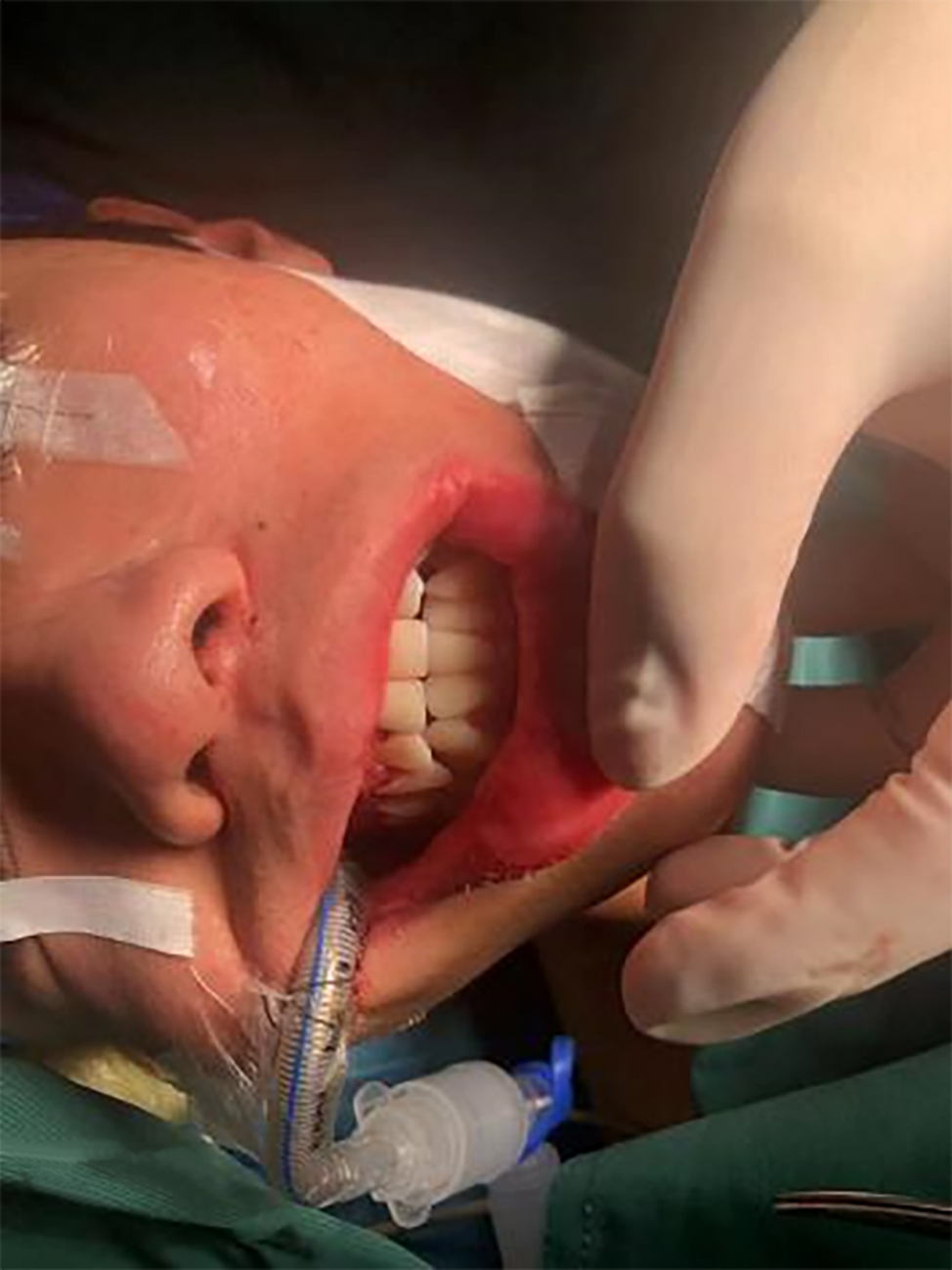
The ETT was pushed back and bent to fit in the right retromolar area and exit from the right corner of the mouth before intermaxillary fixation (Patient 2)

After thoroughly reviewing the literature and actively communicating with the patient, the surgical team decided to adopt retromolar intubation for general anesthesia. The patient took the supine position on the operating table, and routine orotracheal intubation was performed after successful induction of general anesthesia, using a #7.0 reinforced ETT for patient 1 and a #6.0 reinforced ETT for patient 2. Once the airway was established, the ETT was pushed backward, bent, fitted into the retromolar area (left side for patient 1 and right side for patient 2), and allowed to exit from the corner of the mouth (Fig. 3). Intermaxillary fixation was performed customarily after implantation of the intermaxillary traction nail, and the ETT did not interfere with the restoration of the intermaxillary relations (Fig. 4). The ETT was fixed onto the buccal mucosa with 7 − 0 silk suture. For both patients, the surgery was performed as planned without complications, and the intermaxillary fixation was removed at the end of the surgery. The occlusal relations were well restored, and the mouth could open and close normally. The silk suture fixing the ETT was then cut off, and the ETT was moved back to the center of the mouth. The emergence from general anesthesia and the removal of ETT were all unremarkable. During the surgery, there were no signs of airway compression in the routine anesthesia monitoring.

## Methods

Maxillofacial fractures alter facial structures and may also affect the size of the retromolar area, but the existing literature does not indicate if the retromolar area of maxillofacial fracture patients can accommodate a standard reinforced ETT. We thus conducted a retrospective survey using 54 clinical cases of maxillary fracture, mandibular fracture, or complex fracture of multiple maxillofacial bones. These cases were from 38 male and 16 female inpatients hospitalized from January 1, 2020 to September 1, 2022. The basic data included age, sex, and type of fracture (maxilla, mandible, complex). All cases also included the 3D CT data, collected at the coronal position, that were used to determine the presence of the third molars and the height and width of the retromolar area on both sides.

Maxillofacial imaging was collected on a SOMATOM Force CT scanner (Siemens, Germany) with CARE Dose4D automatic tube current modulation (tube voltage 120 kV, quality reference mAs, 250). The slice thickness and slice interval were both 2 mm. The scan range was from the roof of the frontal sinus to the base of the mandible. The cross-sectional and coronal images were reconstructed using the convolution kernels Qr36 and Qr59, respectively, for both the bone window and the soft tissue window. The VR images were reconstructed using MM Reading in Syngo.via (VB10). The presence of the third molars were then determined, and the retromolar areas were measured as described above.

Statistical analyses were carried out using Stata 17.0 (StataCorp, College Station, TX, USA) to evaluate the correlation between the retromolar areas and sex, fracture type, and the presence of third molars. The difference between two groups was analyzed by Student’s t-test, and the difference between multiple groups was assessed by one-way analysis of variance (ANOVA). Data were expressed as mean ± standard deviation (SD) and deemed significantly different when *P* < 0.05. The dimensions of the retromolar areas (left height, LH; left width, LW; right height, RH; right width, RW) were also compared with ETT size.

## Results

Table 1 summarizes the aggregate data of the patients, and Table S1 gives the detailed information of each patient. Of the 54 patients, 38 were male and 16 were female. The breakdown of cases according to the type of fracture was as follows: maxillary fracture, 27; mandibular fracture, 22; complex fracture of multiple maxillofacial bones, 5. Among all patients, 36 had at least one third molar on the left side and 18 did not have a third molar on the left side, while 38 had at least one third molar on the right side and 16 did not have a third molar on the right side. The dimensions (mm) of the retromolar areas were as follows: male, LH = 9.39 ± 1.77, LW = 12.08 ± 0.98, RH = 9.81 ± 2.23, RW = 11.77 ± 1.08; female, LH = 8.82 ± 1.53, LW = 10.51 ± 1.00, RH = 9.73 ± 1.60, RW = 10.63 ± 1.58.

**Table 1:** Clinical characteristics of the surveyed patients

	Male	Female
Number of patients	38	16
Age (year)^§^	44.1	54.3
Type of fracture (maxillary/mandibular/complex)	18/18/2	9/4/3
Third molar (None/Top/Bottom/All)		
Left Right	9/2/10/17 10/5/7/16	9/1/3/3 6/2/3/5
Retromolar area (height × width)		
Left (mm^2^)	(9.39 ± 1.77) × (12.08 ± 0.98)	(8.82 ± 1.53) × (10.51 ± 1.00)
Right (mm^2^)	(9.81 ± 2.23) × (11.77 ± 1.08)	(9.73 ± 1.60) × (10.63 ± 1.58)

The results showed that compared to the female patients, the male patients had significantly wider retromolar area both on the left (12.08 vs. 10.51, *p* < 0.001) and on the right (11.77 vs. 10.63, *p* = 0.015), but there was no significant difference in the height of the retromolar area on either side. The type of fracture did not cause any statistically significant difference in the retromolar area.

Patients who had at least one third molar on the right side had significantly higher RH than patients who did not have any third molar on the right side (10.41 vs. 8.37, *P* < 0.001), but RW, LH, and LW were indistinguishable. Patients who had at least one third molar on the left side had indistinguishable LH, LW, RH, and RW with patients who did not have any third molar on the left side.

The dimensions of the retromolar areas were then compared against the outer diameter of a series of ETT (Table 2). The routinely used ETT is 7.5 mm (10.3 mm OD) for male patients and 7.0 mm (9.8 mm OD) for female patients, respectively. For male patients, LH was significantly less than 10.3 mm, but LW and RW were both significantly higher than 10.3 mm. For female patients, LH was significantly less than 9.8 mm, but LW was significantly higher than 9.8 mm. For all patients, LW and RW were greater than the outer diameter of the respective typical ETT (10.3 mm for male and 9.8 mm for female), and LH and RH were less than 1 mm smaller than the outer diameter of the respective typical ETT.

**Table 2:** The dimensions of the retromolar areas and the size of endotracheal tubes

ETT (mm)		Male				Female			
ID	OD	LH	LW	RH	RW	LH	LW	RH	RW
7.5	10.3	**<**	**>**	n.s.	**>**	-	-	-	-
7.0	9.8	**<**	**>**	**>**	**>**	**<**	**>**	n.s.	n.s.
6.5	8.9	**>**	**>**	**>**	**>**	**<**	**>**	**>**	**>**
6.0	8.2	**>**	**>**	**>**	**>**	**>**	**>**	**>**	**>**

ETT, endotracheal tube (ID, inner diameter; OD, outer diameter). Retromolar area: LH, left height; LW, left width; RH, right height; RW, right width. <, tube size is less than 1 mm larger; > tube size is smaller; n.s., tube size is not significantly different from the dimension of the retromolar area; -, not compared

## Discussions

Nasotracheal intubation is the standard alternative when orotracheal intubation is contraindicated, but due to the risk of brain damage, it is prohibited for patients with skull base fractures, naso-orbito-ethmoid complex fractures, or undergoing surgeries involving the nose, paranasal sinuses, maxilla, nasolacrimal duct, and transsphenoidal pituitary gland surgery [6, 7]. In these situations, tracheostomy or submental intubation seem to be the only options. Tracheostomy is an invasive procedure with many potentially serious complications [8, 9] and a reported mortality rate of 0.5–2.7% [10]. Other complications include tracheal stenosis, intraoperative secondary bleeding due to injuries of neck blood vessels or the thyroid gland, and infection [11, 12]. The scar from tracheal incision is also unpleasant. Therefore, it is difficult to recommend tracheostomy to patients with isolated facial fractures not requiring long-term airway management. Submental intubation is reported to have a success rate of 100% without major complications [13]. Compared to tracheostomy, it is less timely or costly, and the scar is tolerated better aesthetically. However, it still has a complication rate of about 9%. The most common complications are infection, scarring, and salivary fistula, each accounting for 39.1%, 13%, and 11.9% of all complications, respectively [14]. Other reported complications include damage to the tracheal device, fistula formation, prolapse or obstruction of the right main bronchial tube, hypertrophic scarring, accidental extubation of pediatric patient, excessive bronchial flexion, transient lingual nerve palsy, venous bleeding, oral mucoceles, migration of submental wound dressing [13], sublingual duct injury, and oral cutaneous fistula [15].

Clinicians strive to find a simpler, safer, and easier method to give general anesthesia to patients who have contraindications for oral and nasal intubation. Martinez-Lage [3] performed a semilunar osteotomy at the retromolar area and laid the ETT in the resulting notch. This pioneering approach has since been examined by many researchers and is now considered a viable option to avoid occlusal interference, as it advances the ETT into the retromolar area and then into the pharyngeal cavity, thus bypassing intraoral secretions and intraoral structures such as the tongue. Furthermore, because there are two retromolar areas, the healthy side can be safely used even if the opposite side is involved in a disease. Truong et al. [16] reviewed 311 dental pantomograms to measure the height (from the lowest point of the maxillary tuberosity to the mandibular alveolar ridge) and width (from the last mandibular molar to the junction of the anterior border of the ramus with the mandibular body of the mandible) of the retromolar area. Truong also suggested in an operation technique communication earlier that retromolar intubation “is almost invariably sufficiently large to accommodate a 7.0-mm cuffed tracheal tube” [17].

As a new oral intubation technique, retromolar intubation provides an alternative for maxillofacial trauma patients with contraindications of conventional oral and nasal intubation, even in extreme cases with trismus caused by mouth opening difficulties. Complications that have been reported for retromolar intubation include (1) changes in the anatomical structure of the vertebrae (due to the tension from the arch structure of the ETT impacting the atlantoaxial joint), (2) bleeding, inflammation, and/or infection (e.g., due to semilunar osteotomy), and (3) tube prolapse (due to poor fixation, movement from surgical operation, etc.) [18]. Hence, the oral mucosa through which the ETT passes needs to be protected carefully to prevent damage, and care also needs to be taken to prevent damage to the ETT, as an intermaxillary traction nail is implanted and steel wire is used for intermaxillary ligation during the operation. In addition, to prevent accidents, reinforced ETT needs to be used to keep the airway open, since it can withstand pressure better and is not easily deformed by external forces.

Sittitavornwong et al. [5] calculated the dimensions of the retromolar area by 3D reconstructed scans to assess if reinforced ETTs of different size (6.0, 6.5, 7.0, 7.5, 8.0) could be accommodated, and concluded that the reinforced ETTs of size 6.0, 6.5, and 7.0 could be fitted into the retromolar area without causing occlusal interference in setting intermaxillary fixation. Reiterer et al. [19] studied 100 adult patients with an existing retromolar gap at the right mandible and found that the retromolar approach using a Miller #4 blade provided a better view in laryngoscopy than the conventional method using a Macintosh #3 blade, thus demonstrating an elegant technique for adults with anticipated difficult intubation. Thong and Wong [20] reviewed the advantages of the Bonfils retromolar intubation fiberscope compared to other airway devices and summarized the indications of its use. Togioka et al. [21] used the retromolar space to access the oropharynx for a patient with severe trismus and were able to insert a 6.5 mm ETT behind the left maxillary molar, and then guided the ETT into the pharynx and past the vocal cords with a 5.5 mm diameter flexible video endoscope.

Chen et al. [22] enrolled 36 emergency medicine residents and attending physicians in a randomized crossover manikin study and found that in difficult airway situations such as limited mouth opening and neck rigidity with and without tongue edema, compared to the standard midline approach, the retromolar approach increased the first attempt success rate, reduced the duration of successful intubation, and reduced the self-reported difficulty.

The ideal tube size depends on the purpose and duration of intubation. Magill argued in 1928 for the use of “the largest endotracheal tube that the larynx can comfortably accommodate” [23]. However, Stenqvist et al. [24] investigated the use of smaller tubes in 10 patients with no known airway disease undergoing minor ENT surgery and found that using a smaller tube did not cause high intratracheal pressures, concluding that 6.0 mm and 7.0 mm tubes “can be used safely together with artificial ventilation by flow cycled ventilators during anesthesia.” Although it was still rather often in the 1990s to use a 9.0 or even 10.0 mm tube for men and an 8.0 mm tube for women, in recent years the most used tube size is not bigger than 7.5 mm.

Table 2 shows that the width of the retromolar area was always bigger than the OD of the ETT but the height could fall short by 1 mm. Ueno et al. reported that the average thickness of the oral mucosa measured by CT is 2.83 mm [25], and our calculation of the retromolar area (Sect. 2) already considered a uniform mucosal thickness of 3 mm. Nevertheless, the oral mucosa has similar viscoelastic properties to the skin, and its initial thickness can be compressed by 30–40% [26]. It appears that the retromolar area approach can still be selected as the general anesthesia approach for patients with indications in clinical operations, and a smaller tube always remains an option in special cases. For example, Patient 2 in the case report used a 6.0 mm reinforced ETT (OD 8.4 mm).

## Conclusions

Due to sample size and possible selection bias, the results of this study may have the following limitations: 1, the sample size is relatively small, and the source of data for a single center, could lead to influence the clinical significance of the results, especially for the influence of gender and the fracture types. 2. The post-molar area was measured based on 3D CT scan images, and there was a lack of comparison with other measurement methods. Although the average value of three measurements was used, this may also cause errors in measurement consistency and accuracy. Also, although many comparisons were performed, no adjustment was made for multiple comparisons, so there is a risk of significance by chance.

Although maxillofacial bone fractures may affect the retromolar area, from two successful surgeries and a retrospective analysis of 54 cases, we still find retromolar intubation a valuable and viable solution of airway management for patients who (1) cannot undergo standard oral or nasal intubation for general anesthesia due to contraindications or planned intraoperative occlusal restoration and/or (2) refuse tracheostomy or submental intubation. In all cases, retromolar intubation was feasible regardless of the presence or absence of a third molar.

### Electronic supplementary material

Below is the link to the electronic supplementary material.

Supplementary Material 1

## Data Availability

The datasets used and/or analysed during the current study are available from the corresponding author on reasonable request.
