# Supplementary material for: Retromolar intubation for the general anesthesia of maxillofacial fracture patients
Source: BMC Oral Health. 2024 Jul 15;24:795. doi: 10.1186/s12903-024-04583-1 (PMC11251245; doi:10.1186/s12903-024-04583-1)
Supplement: Supplementary file 1 — Supplementary Material 1 [file 12903_2024_4583_MOESM1_ESM.docx]

**Table S1.** Patient information.

| Patient ID | Admitted in | Sex | Age | Fracture Type | Height | Weight | BMI | Third molars | | | | Retromolar areas (mm) | | | |
| --- | --- | --- | --- | --- | --- | --- | --- | --- | --- | --- | --- | --- | --- | --- | --- |
|  |  |  |  |  |  |  |  | TL | TR | BL | BR | LH | LW | RH | RW |
| 3 | Jan 2020 | M | 40 | Mandible | 180 | 80 | 24.7 | 1 | 1 | 1 | 1 | 14.24 | 12.98 | 11.89 | 12.16 |
| 4 | Mar 2020 | M | 34 | Maxilla | 170 | 86 | 29.8 | 1 | 1 | 1 | 0 | 8.57 | 11.57 | 11.12 | 9.98 |
| 5 | Apr 2020 | F | 51 | Maxilla | 168 | 66 | 23.4 | 0 | 1 | 0 | 0 | 10.49 | 11.23 | 12.56 | 11.43 |
| 6 | Apr 2020 | M | 35 | Maxilla | 175 | 55 | 18.0 | 1 | 0 | 1 | 1 | 14.34 | 12.67 | 13.76 | 11.67 |
| 7 | May 2020 | F | 72 | Maxilla | 168 | 60 | 21.3 | 0 | 0 | 0 | 0 | 11.18 | 12.32 | 12.13 | 12.73 |
| 8 | May 2020 | M | 53 | Maxilla | 178 | 75 | 23.7 | 0 | 1 | 0 | 1 | 10.95 | 12.34 | 12.50 | 13.12 |
| 9 | May 2020 | F | 65 | Complex | 160 | 56 | 21.9 | 0 | 1 | 1 | 1 | 9.49 | 10.60 | 11.65 | 10.60 |
| 10 | Jun 2020 | M | 25 | Maxilla | 178 | 76 | 24.0 | 1 | 1 | 1 | 1 | 7.66 | 11.62 | 7.21 | 10.99 |
| 11 | Jun 2020 | M | 38 | Mandible | 170 | 65 | 22.5 | 1 | 1 | 1 | 1 | 7.63 | 11.41 | 7.80 | 9.86 |
| 12 | Jun 2020 | M | 26 | Mandible | 167 | 61 | 21.9 | 1 | 1 | 1 | 1 | 9.94 | 11.83 | 9.44 | 11.76 |
| 13 | Jul 2020 | F | 68 | Mandible | 158 | 68 | 27.2 | 0 | 0 | 0 | 0 | 9.43 | 9.81 | 8.83 | 9.14 |
| 14 | Aug 2020 | F | 84 | Mandible | 155 | 56 | 23.3 | 0 | 0 | 0 | 0 | 9.10 | 10.89 | 7.80 | 9.11 |
| 15 | Sep 2020 | M | 49 | Maxilla | 178 | 75 | 23.7 | 1 | 1 | 1 | 1 | 9.50 | 13.51 | 7.27 | 11.36 |
| 16 | Sep 2020 | F | 58 | Maxilla | 165 | 63 | 23.1 | 0 | 1 | 1 | 1 | 7.92 | 9.73 | 9.57 | 10.40 |
| 17 | Oct 2020 | F | 56 | Maxilla | 156 | 52 | 21.4 | 0 | 0 | 0 | 0 | 6.46 | 9.23 | 7.62 | 8.46 |
| 18 | Nov 2020 | M | 46 | Maxilla | 175 | 70 | 22.9 | 1 | 1 | 1 | 1 | 9.02 | 11.23 | 8.88 | 10.99 |
| 19 | Nov 2020 | M | 56 | Maxilla | 175 | 85 | 27.8 | 1 | 1 | 0 | 0 | 12.23 | 13.52 | 16.27 | 14.46 |
| 20 | Dec 2020 | M | 64 | Mandible | 163 | 59 | 22.2 | 1 | 1 | 1 | 0 | 9.57 | 12.45 | 13.57 | 13.05 |
| 21 | Dec 2020 | M | 37 | Maxilla | 182 | 75 | 22.6 | 1 | 1 | 1 | 1 | 9.60 | 13.21 | 11.02 | 11.80 |
| 22 | Dec 2020 | M | 53 | Maxilla | 172 | 66 | 22.3 | 1 | 1 | 1 | 1 | 9.49 | 12.97 | 10.10 | 13.85 |
| 23 | Feb 2021 | F | 41 | Maxilla | 155 | 56 | 23.3 | 1 | 1 | 1 | 1 | 7.48 | 10.96 | 8.98 | 11.23 |
| 24 | Mar 2021 | M | 68 | Maxilla | 164 | 50 | 18.6 | 1 | 1 | 1 | 0 | 9.48 | 11.37 | 13.12 | 12.25 |
| 25 | Mar 2021 | M | 32 | Mandible | 178 | 65 | 20.5 | 0 | 0 | 0 | 0 | 7.59 | 13.04 | 7.88 | 12.42 |
| 26 | May 2021 | M | 53 | Complex | 175 | 69 | 22.5 | 0 | 0 | 0 | 0 | 11.64 | 12.27 | 7.81 | 11.91 |
| 27 | Jun 2021 | M | 26 | Mandible | 175 | 75 | 24.5 | 0 | 0 | 0 | 0 | 7.65 | 10.72 | 6.79 | 11.83 |
| 28 | Jun 2021 | M | 49 | Mandible | 167 | 76 | 27.3 | 1 | 1 | 1 | 1 | 11.50 | 11.74 | 11.45 | 11.56 |
| 29 | Jul 2021 | M | 31 | Maxilla | 183 | 65 | 19.4 | 1 | 1 | 1 | 1 | 8.03 | 11.29 | 11.23 | 13.18 |
| 30 | Jul 2021 | M | 26 | Mandible | 186 | 96 | 27.7 | 0 | 0 | 0 | 0 | 9.45 | 11.15 | 8.48 | 12.43 |
| 31 | Aug 2021 | M | 30 | Complex | 171 | 60 | 20.5 | 1 | 1 | 0 | 0 | 9.10 | 10.71 | 10.50 | 11.05 |
| 32 | Aug 2021 | M | 25 | Maxilla | 174 | 53 | 17.5 | 1 | 1 | 1 | 1 | 9.26 | 12.15 | 8.98 | 11.60 |
| 33 | Sep 2021 | F | 54 | Maxilla | 153 | 65 | 27.8 | 0 | 1 | 0 | 0 | 8.46 | 10.40 | 11.13 | 10.69 |
| 34 | Oct 2021 | M | 52 | Maxilla | 175 | 56 | 18.3 | 0 | 0 | 0 | 0 | 7.77 | 12.06 | 7.99 | 10.98 |
| 35 | Nov 2021 | M | 15 | Maxilla | 168 | 50 | 17.7 | 0 | 0 | 0 | 0 | 9.34 | 11.61 | 9.10 | 9.47 |
| 36 | Nov 2021 | M | 38 | Mandible | 172 | 85 | 28.7 | 0 | 0 | 0 | 0 | 10.69 | 12.25 | 7.84 | 11.74 |
| 37 | Nov 2021 | M | 52 | Mandible | 168 | 70 | 24.8 | 0 | 1 | 1 | 1 | 9.24 | 10.68 | 8.71 | 9.98 |
| 38 | Dec 2021 | F | 37 | Maxilla | 153 | 42.5 | 18.2 | 0 | 0 | 0 | 1 | 10.07 | 10.95 | 10.93 | 10.55 |
| 39 | Feb 2022 | F | 63 | Maxilla | 158 | 57 | 22.8 | 0 | 0 | 0 | 0 | 9.47 | 9.99 | 8.65 | 10.74 |
| 40 | Apr 2022 | M | 75 | Maxilla | 174 | 62 | 20.5 | 0 | 1 | 1 | 1 | 8.41 | 11.43 | 11.91 | 10.59 |
| 41 | Apr 2022 | M | 64 | Mandible | 197 | - | - | 0 | 0 | 1 | 1 | 6.93 | 12.92 | 9.57 | 11.36 |
| 42 | Apr 2022 | M | 68 | Mandible | 173 | 75 | 25.1 | 0 | 0 | 1 | 1 | 9.71 | 12.09 | 10.33 | 11.73 |
| 43 | May 2022 | M | 47 | Mandible | 185 | 90 | 26.3 | 0 | 0 | 1 | 1 | 8.74 | 9.58 | 11.66 | 10.89 |
| 44 | May 2022 | M | 74 | Mandible | 175 | 65 | 21.2 | 0 | 0 | 1 | 1 | 9.46 | 12.29 | 8.15 | 12.71 |
| 45 | Jun 2022 | M | 54 | Maxilla | 170 | 75 | 26.0 | 1 | 1 | 1 | 1 | 11.29 | 13.98 | 10.61 | 12.76 |
| 46 | Jun 2022 | M | 76 | Maxilla | 165 | 63 | 23.1 | 0 | 0 | 0 | 0 | 7.05 | 14.48 | 7.36 | 12.41 |
| 47 | Jul 2022 | F | 29 | Maxilla | 155 | 50 | 20.8 | 0 | 0 | 1 | 1 | 6.92 | 9.85 | 7.94 | 8.72 |
| 48 | Jul 2022 | F | 52 | Complex | 152 | 66 | 28.6 | 0 | 0 | 0 | 1 | 8.11 | 9.96 | 8.94 | 11.74 |
| 49 | Jul 2022 | M | 17 | Mandible | 172 | 63 | 21.3 | 0 | 0 | 1 | 0 | 8.81 | 12.21 | 8.16 | 10.62 |
| 50 | Jul 2022 | F | 27 | Mandible | 155 | 80 | 33.3 | 1 | 1 | 1 | 1 | 7.63 | 9.80 | 8.85 | 9.85 |
| 51 | Aug 2022 | M | 21 | Mandible | 183 | 106 | 31.7 | 1 | 1 | 1 | 1 | 9.69 | 11.68 | 11.00 | 12.26 |
| 52 | Aug 2022 | M | 38 | Maxilla | 173 | 90 | 30.1 | 0 | 0 | 1 | 1 | 8.47 | 11.88 | 8.79 | 11.47 |
| 53 | Aug 2022 | F | 58 | Mandible | 165 | 62 | 22.8 | 1 | 0 | 1 | 0 | 7.37 | 9.53 | 9.12 | 9.98 |
| 54 | Aug 2022 | F | 54 | Complex | 165 | 65 | 23.9 | 1 | 1 | 0 | 1 | 11.60 | 12.84 | 11.00 | 14.71 |
| 55 | Aug 2022 | M | 35 | Mandible | 175 | 95 | 31.0 | 0 | 0 | 1 | 0 | 6.42 | 12.66 | 7.39 | 12.57 |
| 56 | Aug 2022 | M | 54 | Mandible | 172 | 80 | 27.0 | 0 | 0 | 1 | 1 | 8.46 | 11.66 | 7.13 | 12.36 |
